# Perianal Crohn’s Disease in Inflammatory Bowel Disease: Diagnosis, Assessment and Treatment

**DOI:** 10.3390/life16010182

**Published:** 2026-01-22

**Authors:** Ilaria Faggiani, Isabel Lagos Villaseca, Ferdinando D’Amico, Federica Furfaro, Alessandra Zilli, Sara Massironi, Tommaso Lorenzo Parigi, Virginia Solitano, Clelia Cicerone, Laurent Peyrin-Biroulet, Silvio Danese, Mariangela Allocca

**Affiliations:** 1 Department of Gastroenterology and Endoscopy, IRCCS San Raffaele Hospital and Vita-Salute San Raffaele University, 20132 Milan, Italy; faggiani.ilaria@hsr.it (I.F.); damico.ferdinando@hsr.it (F.D.); furfaro.federica@hsr.it (F.F.); zilli.alessandra@hsr.it (A.Z.); massironi.sara1@hsr.it (S.M.); parigi.tommaso@hsr.it (T.L.P.); solitano.virginia@hsr.it (V.S.); cicerone.clelia@hsr.it (C.C.); sdanese@hotmail.com (S.D.); 2 Department of Gastroenterology, Clinica Las Condes, Santiago 7550000, Chile; ilagos@clinicalascondes.cl; 3 Department of Gastroenterology, INFINY Institute, INSERM NGERE, CHRU Nancy, F-54500 Vandoeuvre-lès-Nancy, France; peyrinbiroulet@gmail.com

**Keywords:** perianal disease, Crohn’s disease, inflammatory bowel disease

## Abstract

Perianal fistulizing Crohn’s disease (pfCD) represents one of the most challenging manifestations of CD, often associated with severe phenotypes, refractory luminal inflammation, and a substantial reduction in quality of life. Its pathogenesis is multifactorial and incompletely understood, involving genetic susceptibility, epithelial and stromal dysfunction, and microbiome-related mechanisms. Diagnosis and monitoring rely on advanced imaging, while management requires coordinated medical–surgical strategies. Significant unmet needs persist regarding standardized treatment targets, optimal imaging follow-up, and personalized therapeutic pathways. In this review, we aim to summarise and provide a comprehensive overview of the most recent evidence across pathogenesis, diagnosis, classification systems, and therapeutic approaches in pfCD. We highlight key advances in understanding epithelial–mesenchymal transition, immune–microbiome interactions, and genetic determinants of disease behaviour. Improvements in diagnostic modalities—including MRI-based scores, ultrasound technologies, volumetric assessment, and AI-enhanced imaging—are discussed alongside modern classification systems such as TOPClass. Evidence guiding medical therapy, seton management, and surgical decision-making is reviewed, emphasising integrated, goal-oriented care. Despite substantial progress, pfCD remains a difficult-to-treat condition with persistent gaps in early diagnosis, objective monitoring, and individualized management. Emerging imaging technologies, standardized treatment targets, and structured classification frameworks offer promising strategies to overcome current limitations and improve long-term outcomes.

## 1. Introduction

Perianal Crohn’s disease (pfCD) represents a frequent and burdensome complication of CD, with epidemiological data demonstrating substantial variability across Western populations. In adult cohorts, the prevalence of perianal fistulas ranges widely—from approximately 1.5% to 55% in European studies and 3.3% to as high as 81% in North American referral populations. Incidence estimates are more consistent, with cumulative rates of 8.3% to 11% at one year following CD diagnosis, and long-term European population-based data indicating a cumulative incidence of 21% to 24% over 10 to 25 years. Among patients with perianal involvement, complex fistulas predominate, accounting for roughly 70% of cases across European and US datasets [1].

Adult pfCD cohorts show a male predominance, with a median of 56% male patients, and a median age near 37 years, underscoring the tendency of pfCD to affect individuals early in their disease course. These epidemiological patterns highlight the significant and persistent clinical impact of pfCD and justify ongoing efforts to refine its diagnosis, classification, and management [1].

Despite advances in diagnostic imaging, surgical techniques, and biologic therapies, the optimal management of pfCD remains complex and requires a multidisciplinary approach. Early and accurate diagnosis of pfCD is crucial for preventing irreversible tissue damage and optimizing outcomes.

This review aims to summarize current evidence and evolving strategies for the diagnosis, classification, and treatment of PCD, highlighting recent advances in imaging, biologic and regenerative therapies, and surgical techniques, with the goal of optimizing patient-centered outcomes.

## 2. Materials and Methods

We conducted a comprehensive search of the PubMed, Embase, and Scopus databases up until 31 May 2025, with the aim of identifying studies regarding perianal disease in Crohn’s disease. However, we ensured that our review included the latest relevant publications available up to 20 December 2025. To achieve this, we employed specific search terms such as “perianal disease”, “penetrating disease”, ”fistulizing disease”, “perianal abscess” and “perianal fistula”, in conjunction with “Crohn’s disease”, “inflammatory bowel disease”, “CD”, and “IBD”. We limited our search to articles published in the English language.

Our screening process involved two independent reviewers (I.F. and I.L.V.) who initially assessed titles and abstracts to identify potentially relevant studies, with the screening process organized by thematic areas: diagnostic/imaging studies and therapeutic/interventional studies. Subsequently, we examined the full texts of these selected articles to determine their eligibility for inclusion. Additionally, we manually scrutinized the reference lists of these articles to ensure that no relevant studies were overlooked during the electronic search. The final inclusion of abstracts and articles was based on their relevance and their contribution to the aims of the review.

## 3. Results

### 3.1. Pathogenesis

The pathogenesis of pfCD is multifactorial and remains incompletely understood, with genetic susceptibility, dysregulated cellular signaling, and alterations of the gut microbiome all likely contributing to disease development.

Emerging evidence indicates that pfCD has a partially distinct genetic background from luminal CD. While most CD-associated loci involve genes regulating host–microbial interactions and epithelial barrier function, several variants appear uniquely enriched in perianal disease. These genetic differences may contribute to the poorer response of pfCD to biologics primarily developed for luminal inflammation [2]. Variants in PRDM1, IL-23R, NOD2 (rs72796353), and ATG16L1 have been linked to internal fistulas, highlighting the role of altered innate immunity and autophagy pathways. In contrast, PUS10 polymorphisms may confer protection against perianal involvement [3].

Another relevant mechanism in pfCD is epithelial–mesenchymal transition (EMT), in which epithelial cells lose their characteristic epithelial features and acquire mesenchymal, migratory properties [4]. This process involves downregulation of adhesion molecules such as E-cadherin and upregulation of EMT-associated transcription factors, including SNAIL1, SLUG, and β6-integrin, which promote cell invasion and tissue remodeling. Pro-inflammatory mediators—TNF from immune cells and TGF-β and IL-13 from fibroblasts—are key drivers of EMT activation in fistula tracts [5]. Matrix metalloproteinases (MMPs), produced mainly by mononuclear cells and fibroblasts, also contribute to fistulisation by degrading extracellular matrix components and promoting tissue remodeling and EMT [6].

**Figure 1:**
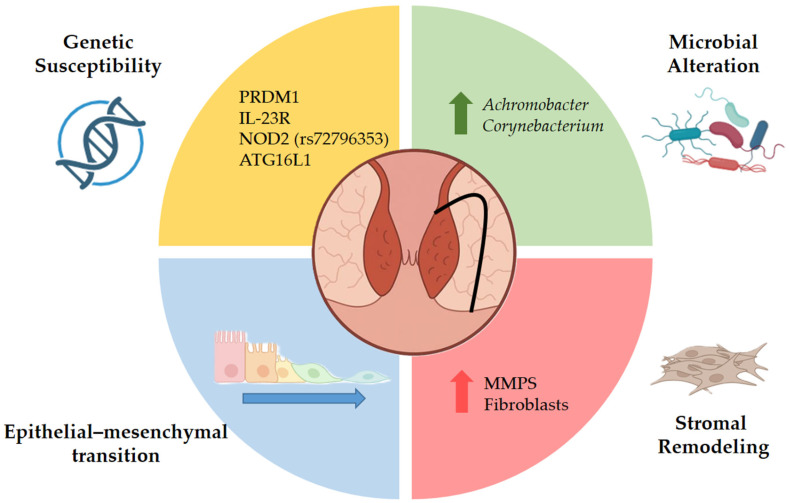
Representation of the multifactorial etiopathogenesis of perianal fistulizing Crohn’s disease (pfCD). The figure illustrates the convergence of genetic susceptibility, epithelial dysfunction with epithelial–mesenchymal transition (EMT), stromal remodeling, and microbial factors in the development and persistence of perianal fistulas. These interrelated pathways contribute to impaired barrier integrity, chronic inflammation, extracellular matrix remodeling, and fibrosis, ultimately sustaining fistula formation and disease chronicity. PRDM1—PR/SET Domain 1 (also known as BLIMP-1, B lymphocyte–induced maturation protein 1); IL-23R—Interleukin-23 Receptor; NOD2 (rs72796353)—Nucleotide-binding Oligomerization Domain-containing protein 2 (specific single-nucleotide polymorphism rs72796353); ATG16L1—Autophagy Related 16 Like 1; MMPs—Matrix Metalloproteinases.

On the other hand, evidence on the microbiome’s contribution to CD-associated fistulas remains limited, but available data suggest that these lesions harbor a microbial profile distinct from cryptoglandular fistulas. Recent 16S rRNA sequencing studies, using new sequencing techniques, have demonstrated that pfCD is associated with higher levels of Achromobacter and Corynebacterium in fustula tract [7]. These populations may contribute to the progression and maintenance of perianal fistulas (Figure 1).

Regarding the latest developments in this field, McGregor et al. used spatial transcriptomics and single-cell technologies to map intestinal fistulas and identified specialized fibroblast subsets—FAS cells—as key drivers of fistula initiation, maintenance, and progression. These cells form structured niches around tracts, promote (extracellular matrix) ECM remodelling and fibrosis, and interact with SPP1^+^ macrophages to sustain chronic inflammation and tissue invasion. Their findings suggest that aberrant developmental and epigenetic reprogramming shapes FAS cell pathogenicity, highlighting these fibroblast states as promising but complex therapeutic targets [8]. In parallel, recent work by Murthy et al. using patient-derived rectal organoids has provided new insights into epithelial contributions to pfCD. Single-cell transcriptomic profiling of organoids and matched mucosal biopsies revealed disease-specific alterations in metabolic, epigenetic, and proliferative pathways within epithelial stem and transit-amplifying compartments, while also preserving ancestry- and sex-related transcriptional signatures. Notably, these organoids lacked the inflammatory signals present in vivo, suggesting that intrinsic epithelial programs may prime disease susceptibility, whereas full pathogenic expression likely requires immune and microbial cues [9].

### 3.2. Classifications

The most commonly used classification of pfCD remains Park’s classification (1976), which provides a detailed anatomical description of the fistula tract in relation to the external anal sphincter and the levator plate [10]. Specifically:

- Intersphincteric: the tract crosses the intersphincteric space but does not traverse the external sphincter
- Transsphincteric: the tract crosses the internal and external sphincters through the intersphincteric space, extending into the ischioanal fossa
- Suprasphincteric: the tract ascends within the intersphincteric space, curves over the puborectalis muscle and through the levator ani into the ischiorectal fossa, and then reaches the perineal skin
- Extrasphincteric: the tract runs from the perineal skin through the ischiorectal fossa and the levator ani to reach the rectum, without involving the internal sphincter

Beyond Park’s anatomical framework, several other classifications are used, including morphology-based systems (e.g., St. James’s University Hospital classification) and those incorporating fistula activity or treatment response (PDAI, and combined activity–morphology scores such as the modified Van Assche index and MAGNIFI-CD) [11,12,13,14].

**Figure 2:**
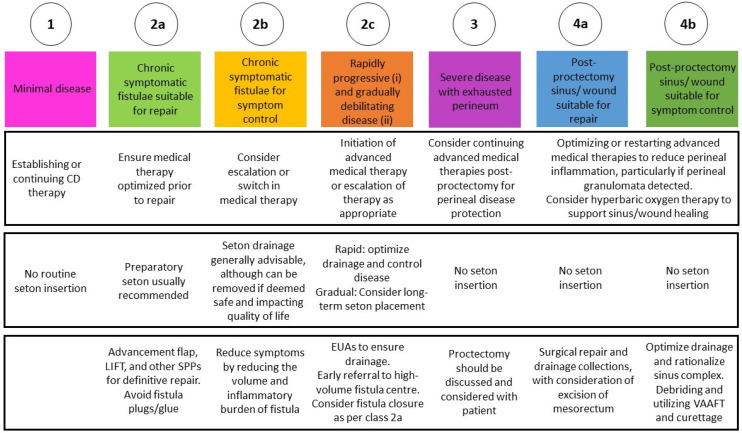
TOP classification for perianal Crohn’s disease (pfCD). The system stratifies patients into eight classes (1, 2a, 2b, 2c, 3, 4a, 4b) based on disease severity, fistula complexity, and therapeutic intent (repair vs. symptom control). For each class, the figure outlines recommended approaches to medical therapy, seton management, and surgical strategies, supporting a structured, goal-oriented, and combined medical–surgical decision-making pathway.

More recently, the TOP classification has emerged as a novel framework that redefines how pfCD is approached. Rather than focusing solely on anatomy or fistula activity, TOP integrates disease severity, prognosis, and therapeutic goals, placing equal emphasis on patient-relevant outcomes and clinical priorities. This model promotes a proactive, coordinated medical–surgical strategy and represents a shift toward a truly goal-oriented, individualized management paradigm for pfCD. Figure 2 summarizes the recommendations for pfCD based on TOP classification [15].

### 3.3. Diagnosis and Assessment

Diagnosis of pfCD should be suspected in patients with CD who present with perianal bleeding, discomfort, or purulent discharge, particularly when associated with fever and pain in the perianal region. In such cases, in addition to a digital rectal examination, both endoscopic assessment of luminal disease and appropriate imaging are indicated. On the other hand, approximately 10% of patients have asymptomatic fistulae, most of which are simple; however, even these individuals carry an increased risk of progressing to symptomatic disease (HR 3.06; 95% CI 1.01–9.27; *p* = 0.048) [16]. Examination under anesthesia (EUA) remains a cornerstone in the diagnosis and management of pfCD. Performed alongside imaging, EUA offers the most accurate assessment of fistula anatomy through systematic digital examination, internal inspection, and targeted palpation. Adjuncts such as dilute hydrogen peroxide may be instilled to highlight internal openings or complex extensions [17]. Moreover, newer minimally invasive techniques—such as video-assisted anal fistula treatment (VAAFT)—enhance EUA by providing both improved visualization and potential therapeutic intervention within the same procedure [18]. Accurate diagnosis of pfCD requires a multidisciplinary approach, integrating gastroenterologists, colorectal surgeons, radiologists, and IBD nurses, as no single modality can fully characterize fistula anatomy, activity, and associated complications. In fact, in a multicenter cohort, multimodality treatment reduced the need for radical surgery (OR 0.21, 95% CI 0.05–0.81) and was predictive of avoiding repeat surgery (OR 0.35, 95% CI 0.17–0.57) [19].

#### 3.3.1. Proctosigmoidoscopy

Long-term cohort studies indicate that rectal involvement not only increases the risk of developing perianal fistulizing CD but also confers a worse prognosis once perianal disease is established [20]. Colonic disease location has been independently associated with the development of pfCD (HR 2.0; 95% CI, 1.01–3.92), while penetrating disease behavior further amplifies this risk (HR 5.65; 95% CI, 2.65–12.03) [20]. Importantly, in patients with established pfCD, concomitant proctitis is linked to more severe outcomes, including a higher likelihood of proctectomy (OR: 3.76, 95% CI: 1.09–12.96, *p* = 0.03) [19]. For this reason, endoscopic assessment with proctosigmoidoscopy is usually recommended before initiating therapy, to accurately evaluate rectal involvement and guide appropriate treatment selection [21].

#### 3.3.2. Pelvic Magnetic Resonance Imaging

Even though pelvic MRI has long been regarded as the gold standard for evaluating pfCD—particularly in deep or complex fistulae—the most recent ECCO–ESGAR-ESP-IBUS guidelines recommend Contrast-enhanced images using intravenous gadolinium for pelvic MRI or EUS as appropriate first-line modalities for initial assessment, followed by EUA [22]. MRI allows detailed mapping of fistula tracts in multiple planes and can identify associated complications such as abscesses. However, it is a costly imaging modality and is less suitable for repeated assessments. Meta-analyses and comparative studies consistently report high sensitivity (typically 80–100%) and specificity above 80% for MRI in detecting perianal fistulas [23]. The presence of rectal inflammation, multiple branched fistula tracts, and associated abscesses favors a CD–related fistula rather than a simple cryptoglandular fistula [24]. The most widely used scoring system to evaluate perianal disease activity is the MAGNIFI-CD index, which incorporates several key parameters: fistula complexity, tract length and sphincter involvement, tissue characteristics (fibrotic vs. purulent), hyperintensity of the primary tract on post-contrast T1-weighted images, and the presence of an inflammatory mass [14].

The recent Delphi consensus by the TOpClass group addressed an important unmet need in pfCD by providing a standardized definition of radiological healing on pelvic MRI. A radiologically healed fistula is primarily characterized by the absence of T2-weighted hyperintense signal within the tract; however, the combination of T2 signal loss with a fully fibrotic (scar-only) tract and/or absence of contrast enhancement provides greater diagnostic confidence. Notably, the MAGNIFI-CD score has demonstrated excellent diagnostic accuracy, with an AUC of 0.95 (95% CI: 0.89–1.00) for predicting long-term clinical closure [25,26]. In the recent external validation of the MAGNIFI-CD score, a reduction of at least 2 points was identified as the optimal threshold for clinical response, whereas a MAGNIFI-CD score of 6 or below on follow-up MRI was established as the criterion for radiological remission [27].

The consensus also proposes recommendations regarding timing, suggesting that radiological healing should be assessed within a flexible window of 6 to 12 months after definitive surgery or initiation of advanced therapies. This range accommodates differences in clinical resources and the varied indications for follow-up imaging [25].

#### 3.3.3. Transrectal Ultrasonography (TRUS)

TRUS is an imaging modality that avoids radiation and contrast exposure. Its low cost and bedside feasibility make it particularly suitable for serial monitoring. TRUS provides high-resolution, circumferential visualization of the anal canal with the patient in the left lateral position, allowing accurate assessment of sphincter anatomy and fistula tracts [28].

In a 2012 meta-analysis, TRUS demonstrated a sensitivity of 0.87 (95% CI: 0.70–0.95), comparable to MRI, but a lower specificity of 0.43 (95% CI: 0.21–0.69) [29]. Technical advances such as hydrogen peroxide–enhanced TRUS have significantly improved diagnostic performance. Hydrogen peroxide instillation markedly enhances internal opening detection, identifying it in over 90% of cases with high concordance to intraoperative findings [30].

Although TRUS excels at detecting collections and delineating tracts within its focal range, it is less reliable for supralevator or very lateral extensions. Contraindications are primarily severe anal stenosis or acute pain that prevents probe insertion.

#### 3.3.4. Transperineal Ultrasound (TPUS)

TPUS is a non-invasive, real-time, bedside imaging technique. TPUS is performed using a high-frequency linear or mini convex probe placed externally on the perineal skin, typically with the patient in left lateral position. Unlike TRUS, TPUS does not require patient preparation such as enemas or the injection of contrast agents, making it particularly suitable in emergency settings when a perianal abscess is suspected [31]. TPUS is therefore well suited for rapid screening, initial assessment, and serial follow-up, and can be complemented by color Doppler imaging to evaluate inflammatory activity [31]. TPUS demonstrates moderate to high concordance with MRI (κ = 0.78 to >0.83) [32]. The most recent meta-analysis, presented as an abstract at ECCO 2025 and including 13 studies in CD patients, reported pooled sensitivities of 96.6%, 99.9%, and 85.5%, with corresponding accuracies of 85.2%, 86.5%, and 83.4% for fistula detection, fistula classification, and abscess detection, respectively [33].

#### 3.3.5. Other Imaging Modalities and Future Perspectives

Several non-traditional imaging modalities have been explored as alternative options for assessing pfCD when MRI, TRUS, or TPUS are unavailable or contraindicated. CT fistulography and CT pelvis may be helpful in acute or resource-limited settings, particularly for detecting abscesses, but remain inferior to MRI for delineating fistula anatomy [34]. FDG-PET/CT can identify metabolically active inflammation and shows early promise, though its clinical utility is constrained by cost, radiation exposure, limited availability, and insufficient validation [35].

Advanced imaging approaches are increasingly enhancing the diagnostic and interventional management of pfCD. Early studies using three-dimensional MRI modelling in small patient cohorts demonstrated improved localization of internal fistula openings, facilitating targeted seton placement and abscess drainage [36]. More recently, three-dimensional transrectal ultrasonography (3D-TRUS) has shown excellent diagnostic performance, with an AUC of 0.934 (95% CI: 0.883–0.988) for detecting key fistula features including number of tracts, echogenicity, tract length, collections, and intrafistular gas [37]. In parallel, AI-based MRI analysis using deep learning models has demonstrated very high accuracy in differentiating pfCD from cryptoglandular fistulas, achieving an AUC of 0.962 (95% CI: 0.903–0.990) and outperforming expert radiologists in a large cohort of 1054 patients (all *p* ≤ 0.001) [38].

### 3.4. Medical Therapy

Antibiotics

Antibiotics such as metronidazole (10–20 mg/kg/day orally for 4–8 weeks) or ciprofloxacin (500 mg orally twice daily for 4–8 weeks) are commonly used as initial therapy for simple perianal fistulas and to control perianal sepsis. However, antibiotics alone rarely eliminate the need for surgical drainage when an abscess is present [39]. Also, durable fistula healing is uncommon with antibiotic therapy alone, and relapse frequently occurs after treatment discontinuation [40]. In fact, evidence—including the ADAFI trial and subsequent studies—shows that combining antibiotics with anti-TNF therapy enhances early clinical response compared with biologic therapy alone [41].

Immunomodulators

Thiopurines (azathioprine and 6-mercaptopurine) or methotrexate may be used as adjuncts to anti-TNF agents to enhance durability of response and reduce immunogenicity. As monotherapy, however, their efficacy is limited, with low rates of fistula closure and high relapse rates after drug withdrawal. In clinical practice, the combination of immunomodulators with anti-TNF agents is often recommended to optimize treatment outcomes [42].

Calcineurin Inhibitors

Tacrolimus has shown encouraging though limited evidence in cases refractory to anti-TNF therapy. While its oral formulation may improve fistula drainage, it rarely induces complete closure and is constrained by adverse effects and the lack of robust, controlled trials [43]. Small randomized and observational studies suggest that topical (0.1% ointment) may improve fistula drainage and perianal ulceration, with minimal systemic absorption and favorable safety profile; however, its impact remains modest [44].

Earlier uncontrolled case series suggested that intravenous cyclosporine could induce rapid symptomatic improvement in refractory perianal disease [45]. However, these responses were typically not durable, with high relapse rates after discontinuation. Additionally, its considerable toxicity profile and increased risk of infections further constrain its clinical use. As a result, cyclosporine is not recommended by major guidelines for routine management of pfCD [40].

TNF Antagonist

The cornerstone of pharmacological intervention for pfCD continues to be TNF antagonist therapy. The efficacy of infliximab in pfCD has been well documented, with approximately 50% of patients achieving a clinical response—defined as at least a 50% reduction from baseline in the number of draining fistulas at consecutive visits four or more weeks apart—and around 36% achieving complete response, defined as the absence of drainage [46]. Similarly, fistula healing rates with adalimumab, based on absence of drainage, are reported at approximately 35% [47]. Consistent with these findings, a recent meta-analysis including six studies demonstrated that adalimumab was not inferior to infliximab in terms of effectiveness for the treatment of pfCD [48]. Early initiation of anti-TNF therapy, particularly in patients with active perianal disease, has been associated with superior outcomes, including reduced risk of developing new fistulas and higher likelihood of achieving sustained remission [49]. Therapeutic drug monitoring has become a key component of pfCD management, as higher anti-TNF trough levels are consistently associated with greater rates of fistula healing. Specifically, a post hoc analysis of the ACCENT-II trial identified that a week 14 infliximab trough concentration of ≥7.2 μg/mL was associated with composite remission, while higher concentrations at earlier time points (≥20.2 μg/mL at week 2, ≥15 μg/mL at week 6) were also predictive of favorable outcomes [50]. In a large multicenter cohort, higher infliximab and adalimumab levels were significantly associated with improved MRI outcomes, characterized by reductions in the inflammatory Van Assche Index subscores and increased rates of radiologic healing and remission [51]. Parallel evidence from a recent multinational consortium demonstrated that higher adalimumab concentrations independently predicted complete fistula healing, with an optimal therapeutic threshold greater than 12.1 μg/mL during maintenance therapy [52].

The timing of TNF antagonist initiation after surgical drainage is crucial, as delays longer than 6 weeks may negatively impact fistula closure rates [53]. Current recommendations from the ECCO and AGA suggest early initiation of anti-TNF treatment after surgical intervention to enhance the likelihood of achieving fistula closure [40]. Evidence from the PISA program further supports the role of combined medical–surgical strategies in pfCD management. Although the original PISA trial was terminated early due to futility and did not yield definitive fistula closure rates, it demonstrated that chronic seton drainage alone was inferior to both anti-TNF therapy and surgical closure in terms of re-intervention rates [54]. Subsequently, the PISA-II study showed that short-term anti-TNF therapy followed by surgical fistula closure resulted in significantly higher rates of radiological healing on MRI (42%) compared with anti-TNF therapy alone (18%) at long-term follow-up (median 5.7 years; *p* = 0.014). Importantly, radiological healing—defined as a completely fibrotic fistula tract on MRI or a MAGNIFI-CD score of 0—was strongly associated with durable outcomes, as no recurrences occurred in patients achieving radiological healing, whereas recurrence was observed in 36% of patients with clinical closure alone [55] (Table 1).

Ustekinumab (IL-12/23 Inhibitor)

Ustekinumab is a potential therapeutic option for patients with pfCD. Although remission rates are lower than those observed with infliximab, accumulating evidence indicates that ustekinumab can be an effective alternative for patients with pfCD who do not respond to anti-TNF therapies.

A meta-analysis including 396 patients demonstrated that ustekinumab was associated with improved fistula remission, with a pooled relative risk of 1.80 (95% CI 1.04–3.11) [56]. The most robust real-world evidence is provided by the multicenter HEAL study, in which 54% of patients with complex perianal fistulas achieved clinical remission, with a 27% relapse rate over a median follow-up of 27 months and a favorable safety profile [57]. These findings position ustekinumab as a viable treatment for complex pfCD, though still clearly less effective than anti-TNF therapy in inducing and maintaining fistula closure.

Although higher serum ustekinumab concentrations correlate with improved luminal response, current data remain limited to define specific trough targets for perianal outcomes. Concentrations above 3.95 µg/mL predicted fistula remission in a recent real-world cohort [58] (Table 1).

Anti IL-23 Agents (p19 Selective Inhibitors)

Although not yet studied in pfCD-specific clinical trials, selective IL-23 blockade (e.g., risankizumab, guselkumab, mirikizumab) shows promise due to its robust luminal efficacy and mechanistic relevance to fistulizing pathways [59]. In a real-world GETAID study assessing long-term outcomes of risankizumab in CD, 60 patients were considered to have active perianal disease at treatment initiation based on physician judgment. During follow-up, perianal clinical response and remission—both assessed by physician evaluation—were each reported in 20 patients (33%) [59].

Vedolizumab (Integrin α4β7 Inhibitor)

Vedolizumab has shown variable efficacy in treating pfCD. Initial real-world cohorts reported low rates of seton removal (≈15%) and recurrence rates approaching 30% among patients with previously inactive disease [60].

However, the ENTERPRISE trial provided stronger prospective data: 53.6% of patients achieved a reduction in fistula drainage (≥50%), and 42.9% achieved complete closure (100%) by week 30. These results indicate that vedolizumab can be effective when combined with optimal surgical management and careful patient selection [61].

In the HEAL study, vedolizumab achieved clinical remission in 46% of patients with complex perianal fistulas, with a 20% relapse rate and a favorable safety profile. Collectively, vedolizumab represents a reasonable second-line option for pfCD, particularly in patients with concomitant luminal disease responsive to gut-selective therapy [57] (Table 1).

JAK Inhibitors

Upadacitinib is emerging as a promising non-anti-TNF therapy for pfCD. Subgroup analyses from the phase 3 U-EXCEL, U-EXCEED, and U-ENDURE trials demonstrated that resolution of fistula drainage at the end of induction was significantly more frequent with upadacitinib 45 mg compared with placebo (44.7% vs. 5.6%; *p* = 0.003), while higher—but not statistically significant—rates were observed during the maintenance phase. Similarly, closure of external fistula openings occurred more frequently in upadacitinib-treated patients during both induction (22.1% vs. 4.8%; *p* = 0.013) and maintenance phases, with statistically significant improvements observed for both the 15 mg and 30 mg maintenance doses compared with placebo [62]. Results from registration trials are progressively being confirmed by real-world evidence from French and North American cohorts [63,64].

Given its rapid onset of action, oral administration, and consistent performance across multiple endpoints, upadacitinib currently stands out as the most effective advanced therapy for pfCD aside from anti-TNF agents, and is increasingly considered in patients who fail or lose response to anti-TNF therapy (Table 1).

Advanced Combination Therapy

Advanced dual therapy remains an emerging approach that may enhance treatment efficacy, particularly in patients with refractory disease who have failed to respond adequately to monotherapy. Reports from multicenter registries and conference presentations suggest that combinations such as anti-TNF plus ustekinumab or anti-TNF plus vedolizumab may yield improvements in drainage, MRI scores, and overall symptoms. In a prospective study with 33 patients, the combination of anti-TNF agents mainly with ustekinumab, and only 1 patient with upadacitinib, resulted in clinical response—partial closure of the external fistula openings and/or a decrease in pain and discharge under pressure without abscesses—in 48.5% of patients, and in 24.2% complete radiological response, defined as MAGNIFI-CD score of 0. Safety in short-term follow-up appears acceptable, though controlled trials are lacking [65].

The table provides an overview of key clinical trials and real-world studies assessing the efficacy of advanced medical therapies for perianal Crohn’s disease, including infliximab (IFX), adalimumab (ADA), ustekinumab (UST), upadacitinib (UPA), and vedolizumab (VDZ). Magnetic resonance imaging (MRI). Standard deviation (SD) Stem-cell therapy (darvadstrocel; mesenchymal stem cells).

**Table 1:** Summary of the main studies evaluating advanced therapies in perianal Crohn’s disease.

Therapy	Author, Year	Study Design	N of Patients	Intervention	Comparator	Mean Age ± SD/(Range)	Definition of Response (Time Point)	Definition of Remission (Time Point)	Results (Time Point)	Ref.
**Anti-TNF** **α**	Present, 1999 (ACCENT II induction)	Prospective	94	63 Induction IFX (5 or 10 mg/kg)	31 placebo	37.2 ± 11.4	≥50% closure of draining fistulas observed at two or more consecutive study visits based on physical evaluation (week 18)	absence of any draining fistulas at two consecutive visits based on physical evaluation (week 18)	Clinical response 68% (IFX 5 mg/kg) and 56% (IFX 10 mg/kg) vs. 26% placebo (*p* = 0.002 and *p* = 0.02, respectively). Clinical remission 55% (IFX 5 mg/kg) and 38% (IFX 10 mg/kg) vs. 13% placebo (*p* = 0.001 and *p* = 0.04, respectively).	[66]
	Sands, 2004 (ACCENT II maintenance)	Prospective	195	96 IFX (5 mg/kg)	99 placebo	37.7 ± 38.0	≥50% closure in the number of draining fistulas at consecutive visits four or more weeks apart based on physical evaluation (week 54)	absence of draining fistulas based on physical evaluation (week 54)	Response 46% (IFX 5 mg/kg) vs. 23% placebo (*p* = 0.001); Remission 36% (IFX 5 mg/kg) vs. 19% placebo (*p* = 0.009)	[46]
	West, 2004	Prospective	24	11 IFX + ciprofloxacin (500 mg×2 die)	13 IFX + placebo	34.0 (18.0–61.0)	≥50% reduction in the number of draining fistulae based on physical evaluation (week 18)	-	Clinical response 73% (IFX + ABX) vs. 39% (IFX + placebo) (*p* = 0.12).	[39]
	Colombel, 2009 (CHARM)	Prospective (Subgroup)	117	70 ADA 40 mg eow	47 placebo	37.1 ± 11.9	-	absence of drainage based on physical evaluation (week 56)	Remission 30% (ADA) vs. 13% placebo (*p* < 0.05)	[47]
	Dewint, 2014 (ADAFI)	Prospective	70	34 ADA 40 mg eow + ciprofloxacin (500 mg×2 die)	36 ADA 40 mg eow + placebo	34.7 ± 11.0 ADA + ciprofloxacin; 37.3 ± 12.4 ADA + placebo	≥50% reduction in the number of draining fistulas based on physical evaluation (week 12)	absence of drainage based on physical evaluation (week 12)	Fistula response 71% (ADA + ciprofloxacin) vs. 47% (ADA + placebo, *p* = 0.047) Complete fistula closure 65% (ADA + ciprofloxacin) vs. 33% (ADA + placebo, *p* = 0.009)	[41]
	Davidov Y et al., 2017	Retrospective cohort	36	IFX (5 mg/kg)	-	21.0 (18.0–27.0)	cessation or significant improvement of fistula drainage reported by patient or treating physician (week 14)	No visible fistula opening on physical examination (week 30)	Response 70% of patients Remission 47.2% of patients	[67]
	Yarur AJ et al., 2017	Multicenter retrospective	117	IFX	-	39 ± 15	-	absence of drainage from the perianal fistula based on physical evaluation (week 24)	Fistula healing 54% of patients	[68]
	McCurdy JD et al., 2020	Retrospective, single-center	22	IFX and ADA	-	38.0 ± 11.2	closure of at least 50% of external fistula openings with reduction in drainage based on physical evaluation (week 52)	complete closure of all external fistula openings with absence of drainage, sustained for at least two consecutive visits based on physical evaluation (week 52)	Remission 36% of patients and response 54% of patients	[69]
	De Gregorio M et al., 2022	Multicenter retrospective	193	117 IFX and 76 ADA	-	35.0 (30.0–44.6) IFX; 37.5 (25.5–46.0) ADA	inflammatory subscore ≤ 6 on the Van Assche Index assessed by pelvic MRI	inflammatory subscore of 0 on the Van Assche Index assessed by pelvic MRI	radiologic healing 47.0% (IFX) and 44.7% (ADA); radiologic remission 17.1% (IFX) and 15.8% (ADA)	[51]
	Chan M et al., 2023	Retrospective, single-center	155	IFX, ADA and golimumab	-	34.7 ± 12.5	-	absence of drainage from all external fistula openings based on physical evaluation (3, 6, and 12 months)	Fistula closure rates: 32% at 3 months, 41% at 6 months, and 47% at 12 months	[70]
Ustekinumab	Chapuis Biron, 2020 (BioLAP)	Multicenter retrospective	207	UST Mantainance	-	37.7 ± 11.4	-	Treatment success: absence of drainage and no anal ulcers based on physical evaluation(6 months) MRI response: radiologist’s appreciation	Treatment success: 38.5% MRI response: 50%	[71]
	Yao et al., 2023	Retrospective cohort	108	UST q8w or q12w	-	29.2 ± 1.0	Response: decrease of >50% in the number of draining fistulas according to the fistula drainage assessment index based on physical evaluation (week 16/20); Radiological improvement: reduction in the number and volume of fistula, and >10% decrease in the MRI signal	Remission: absence of any draining fistula based on physical evaluation; Radiologic healing: absence of a high-signal track on fat-saturated T2 sequences (week 16/20)	Response: 63%; Remission 40.7%; radiological improvement: 31.4%; radiological fistula healing: 44.8%	[72]
	Casanova, 2024 (HEAL study)	Multicenter retrospective	155	136 UST (16 both UST and VDZ)	35 VDZ (16 both UST and VDZ)	45.0 (35.0–54.0) UST; 46.0 (41.0–54.0) VDZ	Reduction of 50% or more in the number of draining tracts based on physical evaluation (weeks 24)	Clinical remission: no drainage through the fistula upon gentle pressure based on physical evaluation MRI response: absence of activity in perianal fistulous tracts and the absence of local complications Combined remission: clinical + radiological remission (week 24)	Response 65% and remission 36%; MRI response 50%; combined remission 30%	[57]
	Chen et al., 2025	Retrospective cohort	143	UST	-	28.6 ± 9.0	-	Clinical remission: absence of pain/drainage based on physical evaluation (2 years); radiological healing: absence of high signal-intensity trajectories on fat-saturated T2-weighted sequences (52 weeks) Deep remission: clinical+ radiological	Clinical remission: 73.9%; Deep remission: 40.0%	[73]
	Liu et al., 2025	Retrospective cohort	89	UST	-	26.0 (22.0–30.0)	MRI improvement: reduction in the number and volume of fistulas and a decrease in MRI signal intensity of >10%	absence of drainage based on physical evaluation; MRI healing: absence of a high-signal tract on fat-saturated T2 sequences (16/20 weeks and 52 weeks)	Absence of drainage: 66.3% (16/20 weeks), 74.2% (52 weeks); MRI improvement 54.7% and MRI healing: 21.8% (52 weeks)	[58]
Vedolizumab	Chapuis-Biron, 2019	Multicenter retrospective cohort	151	VDZ	-	39.8 (21.0–63.0)	-	Clinical success: no draining fistula at a given visit, and no anal ulcers for primary lesions based on physical evaluation (6 months)	22.5% clinical success	[60]
	Schwartz, 2022 (ENTERPRISE)	Multicenter, randomized, double blind trial	16 VDZ regimen; 18 VDZ + wk10	VDZ 300 mg IV at weeks 0, 2, 6, 14, and 22 (VDZ regimen)	VDZ 300 mg IV at weeks 0, 2, 6, 14, and 22 + week 10 (VDZ + wk10)	34.0 (21.0–59.0)	≥50% closure based on physical evaluation (22 and 30 weeks)	100% closure based on physical evaluation (30 weeks)	Response: 64.3% VDZ and 42.9% VDZ + wk10 Remission: 50% VDZ and 35.7% VDZ + wk10	[61]
	Casanova, 2024 (HEAL)	Multicenter retrospective cohort	155	136 UST (16 both UST and VDZ)	35 VDZ (16 both UST and VDZ)	45.0 (35.0–54.0) UST; 46.0 (41.0–54.0) VDZ	Reduction of 50% or more in the number of draining tracts based on physical evaluation (week 24)	Clinical remission: no drainage through the fistula upon gentle pressure based on physical evaluation MRI response: absence of activity in perianal fistulous tracts and the absence of local complications Combined remission: clinical + radiological remission (week 24)	Response 57%; remission 32%; MRI response 78%; combined remission 22%	[57]
Upadacitinib	Colombel, 2025 (U-EXCEL, U-EXCEED, U-ENDURE)	Post hoc analysis of phase 3 trials	128	UPA 45 mg, 30 mg and UPA 15 mg	42 placebo	33.9 ± 11.7 UPA; 35.7 ± 9.5	resolution of drainage based on physical evaluation (weeks 12 and 52)	closure of all external openings based on physical evaluation (weeks 12 and 52)	Response: 44.7% (week 12, upadacitinib 45 mg; 5.6% placebo), 28.6% (week 52, upadacitinib 15 mg; 0% placebo), 23.1% (week 52, upadacitinib 30 mg; 0% placebo); Remission: 22.1% (week 12, upadacitinib 45 mg; 4.8% placebo), 18.8% (week 52, upadacitinib 15 mg; 0% placebo), 16.0% (week 52, upadacitinib 30 mg; 0% placebo)	[62]

Mesenchymal stem cell therapy (SCT) has shown potential for treating pfCD, and is a promising option for refractory pfCD [74]. SCT may enhance healing and produce a localized anti-inflammatory and regenerative effect [75].

Results from the ADMIRE-CD phase 3 trial showed significantly higher combined remission with darvadstrocel compared with placebo (51.5% vs. 35.6% and 56.3% vs. 38.6% at 24 and 52 weeks, respectively) [76]. The more recent ADMIRE-CD II study did not show a significant difference between treatment groups; notably, the placebo arm exhibited a higher-than-expected response rate, likely influenced by optimized fistula preconditioning prior to injection [77]. Despite these findings, emerging real-world data remain encouraging. In a 2025 multicenter cohort from the Spanish National Health System, combined remission was achieved in 69.2% of patients at 6 months and 57.7% at 12 months, with no treatment-related adverse events reported [78]. These findings reinforce that, when used in appropriately selected patients with optimized surgical conditioning, MSC therapy can deliver clinically meaningful and durable benefits in practice despite mixed results in recent randomized trials.

Autologous adipose-derived stem cells (ADSC) constitute a developing option, offering the advantage of patient-derived cells with minimal immunogenic risk. Preliminary clinical studies have demonstrated that local administration of autologous ADSCs may result in substantial clinical improvement and radiological improvement. Healing rates vary depending on the technique and need for concomitant surgical preparation, but overall outcomes suggest that autologous ADSCs can be safely harvested, expanded, and reinjected to promote fistula closure with a low incidence of adverse events [79].

Hyperbaric Oxygen

Hyperbaric oxygen therapy (HBOT) is a growing area of interest for patients with refractory pfCD, targeting tissue hypoxia and promoting local wound healing. While some small real-world series report symptomatic improvement and occasional healing of perianal tracts, evidence remains inconsistent [80].

The HOT-REVA pilot study specifically evaluated Crohn’s-related rectovaginal fistulas and found no cases of clinical closure at 3-month follow-up, despite HBOT being feasible and generally well tolerated [81]. A 2024 prospective cohort, demonstrated that HBOT can lead to significant reductions in fistula drainage and improvement in clinical outcomes, with 54.5% clinical remission and sustained benefit up to 9 months post-treatment [80]. However, a 2025 series of highly refractory cases found limited objective healing, although most patients reported symptomatic relief [82].

Surgical Therapy

Interdisciplinary care is fundamental in the management of pfCD, with surgical intervention serving a cornerstone that must be closely integrated with optimized medical therapy [42]. Surgical goals include controlling sepsis, preserving sphincter functions, and enhancing the conditions necessary for durable fistula healing [40]. Procedures range from abscess drainage and seton placement to sphincter-sparing techniques such as advancement flaps. Each approach is tailored to the patient’s anatomy, fistula complexity, and degree of inflammation, aiming to improve healing while minimizing recurrence and preserving function [15].

1. Examination under Anesthesia (EUA): guiding surgical strategy [17]
2. Seton placement: balancing stabilizations with healing goals

Setons play a practical role in keeping drainage controlled, lowering the chance of abscesses, and preparing the fistula for later surgical repair. Historically considered indispensable, recent data challenge the assumption that setons improve long-term outcomes. A 2025 multicenter analysis found no significant difference in remission at 6 (OR, 0.81; 95% CI, 0.41–1.59) or 12 months (OR, 0.63; 95% CI, 0.31–1.27) between patients with and without setons after adjustment for fistula complexity [83]. TOpClass now recommends individualized seton strategies: typically avoided in minimal disease (Class 1), used briefly before repair in suitable fistulas (Class 2A), and employed long-term for symptom control in complex or progressive disease (Class 2B–2C) [15].

3. Fistula closure: repairing fistula without compromising function

When anatomy and inflammation permit, definitive repair aims to eliminate the internal opening while preserving continence. This can be achieved through various techniques, including advancement flaps and sphincter-sparing methods, which focus on minimizing sphincter division to reduce the risk of incontinence.

- Endorectal advancement flap (AF): Removes the epithelized tract and covers the internal opening. Variants (mucosal, partial thickness, full thickness) balance recurrence rates with sphincter safety [84].
- Ligation of the intersphincteric fistula tract (LIFT): Approaches the fistula through the intersphincteric plane, minimizing sphincter injury. Healing rates are comparable to AF, and it is preferred when anatomy limits flap mobility [84].
- Fistulotomy: Reserved only for simple, low tracts due to the risk of incontinence; rarely appropriate in CD.
- Novel techniques: Minimally invasive options such as FiLAC (laser closure) and VAAFT (video-assisted treatment) have reported encouraging but variable success. Evidence is limited by small sample sizes and heterogeneous definitions of healing. Availability is mostly restricted to specialized centers [18].
- Biomaterials such as fibrin glue and fistula plugs have shown inconsistent results and are not routinely recommended for PFCD [40].

4. Addressing Advanced Disease: From diversion to proctectomy

Management of pfCD requires thoughtful escalation when symptoms remain uncontrolled or when extensive tissue destruction prevents meaningful repair. Fecal diversion can reduce symptoms and provide temporary control of local infection [85]. In practice, stoma reversal is possible for only a few patients, highlighting that diversion typically functions as a bridge to stabilizing the disease rather than a final solution [85]. In cases of irreversible perineal destruction or exhausted tissue planes (TOpClass Class 3), proctectomy becomes the appropriate intervention. Even after proctectomy, patients may experience persistent perineal sinus tracts, chronic wounds, or pelvic sepsis, complications that align with TOpClass Class 4 and require ongoing management. Care for persistent perineal disease can range from local excision and flap repair to negative-pressure therapy or, for selected patients, hyperbaric oxygen. In these complex scenarios, patients benefit most from a tailored, team-based approach that prioritizes symptom relief, wound healing, and quality of life [15]. Table 2 summarizes the ongoing studies on the treatment of pfCD.

**Table 2:** Ongoing phase 2 and 3 studies on treatment in perianal Crohn’s disease.

NCT Number	Name	Treatment	Phase	Reference
NCT05347095	FUNZION CD	guselkumab	3	[86]
NCT04847739	STOMP-II	Seeded Cells on Matrix Plug	2	[87]
NCT06925594	-	Human TH-SC01 Cell	3	[88]
NCT04010526	ADICROHN2	Autologous ADSVF	2	[89]
NCT06918808	-	DB-3Q	2a	[90]
NCT06059989	DIRECTCD	Subcutaneous infliximab	3	[91]
NCT03901235	MCS	Mesenchymal Stromal Cells	2	[92]

## 4. Discussion

pfCD represents a particularly complex manifestation of CD, yet patients with pfCD remain underrepresented in clinical studies and dedicated trials—a growing concern in the broader conversation on equity in research. This underrepresentation is paradoxical, given that pfCD is far from rare: it may affect up to 80% of patients with CD, frequently presenting with complex and refractory phenotypes [1]. Historically, pfCD has been recognized as a negative prognostic indicator [93], and recent work, including the PROGNOS study, reinforces its role as a key risk factor in predicting disease course [94]. Progress in the field is limited by marked heterogeneity across studies, as underscored by recent systematic reviews and meta-analyses, with variability in design, imaging protocols, and endpoints hindering robust comparisons and standardized treatment strategies [1]. Most evidence comes from retrospective or observational cohorts, and response/remission definitions vary widely, ranging from physician assessment to clinical fistula closure and radiological outcomes, complicating interpretation of treatment efficacy.

On the other hand, the TOPClass initiative represents a major step forward, offering a dynamic, goal-oriented framework that moves beyond static, anatomy-based classification to incorporate clinical context and patient priorities. This model has the potential to enhance consistency in reporting, streamline personalized care pathways, and ultimately improve patient outcomes. Complementing this effort, the same group developed a Delphi-based consensus to define radiological healing on MRI, addressing another long-standing gap in pfCD assessment [25].

Current guidelines recommend MRI and/or TRUS as first-line modalities followed by EUA, while TPUS is emerging as a cost-effective, non-invasive tool increasingly accessible to gastroenterologists [22]. In parallel, the development of AI–based models for MRI interpretation and fistula characterization offers a promising frontier, with early studies demonstrating high accuracy in detecting tracts, internal openings, and abscesses, and the potential to standardize assessments, reduce interobserver variability, and support treat-to-target strategies in pfCD. For example, pTRACK is an AI-assisted platform for pelvic MRI analysis in pfCD that supports automated detection, quantification, and 3D reconstruction of fistula tracts. It provides objective imaging biomarkers, including normalized mean T2 signal intensity and fistula volume, which have shown good diagnostic performance for assessing fistula activity. In a prospective 2025 study, normalized T2 signal intensity achieved an AUC of 0.784 for identifying active pfCD, with 80% overall accuracy and high sensitivity (0.857), performing comparably to the MAGNIFI-CD index. Both fistula volume and T2 signal metrics demonstrated good-to-excellent reliability and were able to discriminate radiological remission from active disease [95].

Despite significant advances in the development of advanced therapies for CD, anti-TNF agents remain the cornerstone of treatment for pfCD. Higher drug exposure and optimized strategies, including dose intensification and subcutaneous formulations, may further enhance therapeutic efficacy in this setting. At the same time, other advanced therapies are being actively investigated, with emerging evidence suggesting promising results for JAK inhibitors and selective IL-23 blockade [86]. In selected patients with refractory or complex disease, advanced combination therapy, involving biologic–biologic or biologic–small molecule combinations, is also being explored as a potential strategy to enhance efficacy and overcome treatment resistance, although safety and long-term outcomes require further evaluation [42]. In parallel, rapid progress in understanding the pathogenic mechanisms underlying pfCD—particularly involving epithelial, stromal, and immune pathways—may facilitate the identification of novel, mechanism-driven therapeutic targets [8]. Recent transcriptomic analyses have identified a TNF-independent fibroinflammatory pathway in pfCD driven by the TL1A–LTα1β2 axis, leading to fibroblast activation, matrix remodeling, and epithelial alterations even in the absence of active rectal inflammation. The persistence of this pathway despite anti-TNF therapy provides a mechanistic rationale for treatment refractoriness and supports TL1A-targeted strategies as a promising therapeutic avenue in pfCD [96].

Beyond advanced medical therapies, adjunctive approaches are being explored for selected patients with pfCD. MSC therapy—particularly ADSCs—has shown clinically meaningful and potentially durable benefits in real-world cohorts when combined with optimized surgical conditioning, despite mixed results in randomized trials [75]. Although evidence remains limited and heterogeneous, MSCs may represent a promising personalized strategy to complement standard medical–surgical management. HBOT has also been associated with symptomatic and short-term improvement, but appears best suited as an adjunct in multidisciplinary care for complex, refractory disease, given uncertain durability [82]. Further high-quality studies are needed to define efficacy, patient selection, and implementation in routine practice.

We propose several directions to address the major unmet needs in pfCD:

- Evolving disease classification beyond purely anatomical frameworks to incorporate molecular, cellular, and imaging-derived phenotypes that better capture biological heterogeneity and disease behavior. AI may facilitate standardized interpretation of endoscopic, histological, and radiological data.
- Defining objective and reproducible endpoints, including standardized clinical, radiological, and biological targets.
- Implementing longitudinal assessment strategies through prospective cohort designs with serial sampling of imaging, tissue, and circulating biomarkers.
- Optimizing and personalizing therapeutic approaches, focusing not only on novel biologics and small molecules but also on maximizing the efficacy of existing treatments. AI-driven decision support, therapeutic drug monitoring, pharmacokinetic–pharmacodynamic modeling, and rational combination therapies may be necessary to address complex pfCD phenotypes and improve long-term outcomes.

## 5. Conclusions

Perianal Crohn’s disease remains a complex and debilitating manifestation of Crohn’s disease, exerting a profound impact on patients’ quality of life and long-term outcomes. The underlying pathophysiology is still not fully understood, limiting the development of consistently effective treatments. The marked heterogeneity across existing studies further complicates interpretation of outcomes and therapeutic decision-making. There is a clear need for homogeneous, well-designed studies with standardized definitions and endpoints to better characterize disease mechanisms and to inform evidence-based, targeted treatment strategies that can ultimately improve care for patients with pfCD.

## Data Availability

No new data were generated or analyzed in support of this research.

## Abbreviations

The following abbreviations are used in this manuscript:

ADA	Adalimumab
ADSCs	Adipose-Derived Stem Cells
AI	Artificial Intelligence
AUC	Area Under the Curve
CD	Crohn’s Disease
CGF	Cryptoglandular Fistula
CT	Computed Tomography
ECCO	European Crohn’s and Colitis Organisation
EMT	Epithelial–Mesenchymal Transition
ESGAR	European Society of Gastrointestinal and Abdominal Radiology
EUA	Examination Under Anesthesia
FDG-PET/CT	Fluorodeoxyglucose Positron Emission Tomography/Computed Tomography
HBOT	Hyperbaric Oxygen Therapy
IBD	Inflammatory Bowel Disease
IFX	Infliximab
IL	Interleukin
JAK	Janus Kinase
MAGNIFI-CD	Magnetic Resonance Index for Fistula Imaging in Crohn’s Disease
MRI	Magnetic Resonance Imaging
MMPs	Matrix Metalloproteinases
MSC	Mesenchymal Stem Cells
pfCD/PFCD	Perianal Fistulizing Crohn’s Disease
PRO	Patient-Reported Outcome
TNF	Tumor Necrosis Factor
TOPClass	Treat-to-Patient-Goal-Oriented Classification
TPUS	Transperineal Ultrasound
TRUS	Transrectal Ultrasound
VAAFT	Video-Assisted Anal Fistula Treatment
